# Temporal Trends in Second-Stage Cesarean Birth in Ontario, Canada, 2012–2021

**DOI:** 10.1097/og9.0000000000000084

**Published:** 2025-06-05

**Authors:** Vanessa Hébert, Sheryll Dimanlig-Cruz, Giulia M. Muraca

**Affiliations:** Department of Health Research Methods, Evidence and Impact and the Department of Obstetrics and Gynecology, Faculty of Health Sciences, McMaster University, Hamilton, the Better Outcomes Registry & Network (BORN) Ontario, Ottawa, Ontario, Canada; and the Clinical Epidemiology Unit, Department of Medicine, Solna, Karolinska Institutet, Stockholm, Sweden.

## Abstract

**OBJECTIVE:**

To characterize trends in the mode of delivery in the second stage of labor, to assess factors associated with second-stage cesarean birth compared with operative vaginal birth, and to evaluate the contribution of these factors to changes in second-stage cesarean birth rates over time among individuals giving birth in Ontario, Canada.

**METHODS:**

This was a population-based cohort study of all individuals in Ontario, Canada (2012–2021), with singleton term pregnancies reaching full cervical dilation. Temporal trends were assessed with the Cochran–Armitage test. Modified Poisson regression was used to obtain crude (relative risk [RR]) and adjusted relative risk [aRR] and 95% CIs and to quantify the contribution of maternal, obstetric, fetal, and neonatal factors to temporal variations in second-stage cesarean birth rates. All analyses were stratified by parity.

**RESULTS:**

Among 806,645 births included, 26.7% of nulliparous and 6.5% of parous individuals had a second-stage cesarean birth or an operative vaginal birth, with ratios of cesarean to operative vaginal birth of 0.24 and 0.16, respectively. Between 2012–2013 and 2020–2021, second-stage cesarean birth rates increased from 4.8% to 5.8% for nulliparous individuals (*P* for trend<.001, RR 1.16, 95% CI, 1.08–1.24) and from 0.8% to 1.0% for multiparous individuals (*P* for trend<.001, RR 1.30, 95% CI, 1.11–1.51), as did ratios of cesarean to operative vaginal birth (nulliparous individuals 0.22–0.27, *P*<.001; multiparous individuals 0.13–0.18, *P*<.001). Factors strongly associated with second-stage cesarean birth included macrosomia, previous cesarean birth, elevated body mass index, and care by a midwife or family physician. After adjustment for maternal and obstetric factors, the increase in second-stage cesarean birth rates was largely attenuated (nulliparous individuals aRR 1.04, 95% CI, 0.97–1.11; multiparous individuals aRR 1.04, 95% CI, 0.89–1.21), with obstetric practice factors accounting for the largest proportion of the increase (nulliparous individuals 46.3% of the 16.0% increase; multiparous individuals 48.6% of the 29.6% increase).

**CONCLUSION:**

Between 2012 and 2021, second-stage cesarean birth rates and ratios of cesarean to operative vaginal birth increased in Ontario, Canada. Changes in obstetric practices were the primary modifiable drivers of this trend.

## Abstract 2

Rates of cesarean birth at full cervical dilation in Ontario, Canada, increased between 2012 and 2021, driven primarily by changes in obstetric practices.

Over the past three decades, global cesarean birth rates have risen steadily^1^ and rates of operative vaginal birth have declined.^2,3^ This decline in operative vaginal births has limited opportunities to develop proficiency, particularly with forceps,^4^ raising concerns about a potential rise in second-stage cesarean births. Second-stage cesarean births are recognized as technically challenging surgical procedures that carry increased risk compared with cesarean birth in the first stage of labor or operative vaginal birth.^5^ In response, obstetric care organizations have proposed strategies to promote vaginal birth, including increased use of operative vaginal birth.^6^ However, the optimal approach to minimize maternal and neonatal morbidity when choosing between a difficult operative vaginal birth or cesarean birth at full dilation remains unclear.

Presently, there is a lack of high-quality evidence to guide these clinical decisions. Population-level estimates of temporal trends in second-stage cesarean births are scarce, with only a few studies, primarily from Europe, examining these trends, most of which predate initiatives to increase operative vaginal births.^7–10^ These studies report general increases in second-stage cesarean birth rates over time, from 0.5–0.9% to 1.2–2.2%. In Canada, where operative vaginal birth rates are higher,^11^ only one other study has explored these trends (1992–2018).^12^ Because practices may vary across regions, replication is valuable to confirm trends and to understand regional differences. This study aims to characterize the distribution and temporal variation in the mode of delivery in the second stage of labor, to evaluate factors associated with second-stage cesarean birth compared with operative vaginal birth, and to estimate the contribution of changes in maternal, obstetric practice, and fetal–neonatal factors on second-stage cesarean birth rates over time.

## METHODS

We conducted a population-based cohort study of all deliveries in the second stage of labor in Ontario, Canada, between April 1, 2012, and March 31, 2021. Singleton term pregnancies (37 weeks of gestation or more) without congenital anomalies identified before delivery were included. Only individuals with Ontario health insurance coverage were included to facilitate data linkage. The study was approved by the Hamilton Integrated Research Ethics Board (2023-16643-C) on February 17, 2024.

Data were obtained from BORN (Better Outcomes Registry & Network) Ontario, the largest perinatal registry in Canada.^13^ These data were linked to the Canadian Institute for Health Information Discharge Abstract Database, which includes clinical information on hospital admissions. The accuracy of BORN and Discharge Abstract Database data has been validated in previous studies.^13,14^ Neighborhood-level material deprivation quintiles were obtained from the Ontario Marginalization Index, a validated index that quantifies area-level marginalization according to census data.^15^ Ontario Marginalization Index data were linked through individual postal codes with the Canadian Census and Postal Code Conversion File Plus 8A1.^16^

Mode of delivery was classified as spontaneous vaginal birth, operative vaginal birth (vacuum, forceps, sequential operative vaginal birth [involving sequential use of vacuum and forceps]), and second-stage cesarean birth. Although planned sequential use of instruments is not recommended in Canada, guidelines indicate that it may be appropriate to consider a change in instrument if the initial instrument was suboptimally applied or limited by technical issues, because this may avoid a difficult cesarean delivery without compounding risk.^17^ All analyses were stratified by parity, categorized as *nulliparous* (zero previous births) or *parous* (more than zero previous births), because of the expected differences in clinical characteristics and risk between parity groups. Observations with missing mode of delivery or parity were excluded.

The primary indication for second-stage cesarean birth was classified into five categories: obstructed labor in the second stage, abnormal fetal heart rate, failed operative vaginal birth, other maternal factors (eg, obstetric complications, health condition, failed induction, maternal request), and other fetal factors (eg, cord prolapse, macrosomia, malposition). Variables potentially associated with mode of delivery included individual characteristics (age, prepregnancy body mass index [BMI, calculated as weight in kilograms divided by height in meters squared], race, material deprivation), comorbidities (preexisting diabetes mellitus, gestational diabetes, preexisting hypertension, hypertensive disorders of pregnancy [preeclampsia, eclampsia, HELLP (hemolysis, elevated liver enzymes, and low platelet count) syndrome], tobacco use in pregnancy, assisted reproductive technology [in vitro fertilization with or without intracytoplasmic sperm injection], previous cesarean birth), obstetric practices (first-trimester visit, intrapartum admitting clinician, neuraxial analgesia in labor, induction of labor, oxytocin use in labor), and fetal–neonatal characteristics (fetal presentation, gestational age, birth weight, congenital anomaly identified after birth). Race was included to account for structural inequalities and potential disparities in health care access and quality that may influence mode of delivery outcomes. Variable definitions and coding are detailed in Appendix 1, available online at http://links.lww.com/AOG/E154.

Descriptive statistics were used to present the distribution of mode of delivery by parity, and the ratio of second-stage cesarean birth to operative vaginal births was calculated.^18^ Temporal trends were assessed with the two-sided Cochran–Armitage test for linear trends in proportions by year, and rates were compared between 2020–2021 and 2012–2013. The distribution of maternal, obstetric, and fetal–neonatal characteristics was compared across delivery modes, including spontaneous vaginal birth, to provide a comprehensive understanding of second-stage births. Standardized differences were calculated for second-stage cesarean birth compared with operative vaginal birth groups, with differences greater than 0.1 indicating imbalances.^19^

For each characteristic, we estimated the crude relative risks (RRs) and adjusted relative risks (aRRs) and 95% CIs for the association between second-stage cesarean birth and operative vaginal birth using modified Poisson regression with robust error variance. To assess the contribution of individual characteristics on second-stage cesarean birth compared with operative vaginal birth, we calculated population attributable fractions using the Miettinen formula.^20^ The population attributable fraction quantifies the fraction of all second-stage cesarean birth cases attributable to a specific exposure. Missing values of 3% or more were handled by including a missing indicator in the regression models. Missing values less than 3% were excluded from multivariable analyses.

To evaluate the contribution of maternal, obstetric, fetal, and neonatal factors to temporal changes in second-stage cesarean birth rates, we used modified Poisson regression to quantify trends by year and estimate the magnitude of change over time (2020–2021 vs 2012–2013). Because changes in maternal characteristics and comorbidities can influence changes in obstetric practices, we used sequential models to assess how each group of factors affected the relationship between period and second-stage cesarean birth. We first calculated unadjusted RRs and 95% CIs, followed by sequential adjustments for individual characteristics (age, prepregnancy BMI, material deprivation), comorbidities (preexisting diabetes, gestational diabetes, preexisting hypertension, hypertensive disorders of pregnancy, smoking, assisted reproductive technology, previous cesarean birth [parous only]), obstetric practices (first-trimester visit, labor induction, intrapartum oxytocin, neuraxial analgesia in labor), and fetal–neonatal factors (fetal presentation, gestational age, birth weight, newborn anomaly) to estimate their contributions to observed trends. We hypothesized that factors responsible for the rise in second-stage cesarean birth would either eliminate or substantially attenuate the observed trend. This analysis was conducted with complete-case analysis.

Generalized estimating equations assuming an exchangeable correlation structure were used in all models to adjust for the potential nonindependence of deliveries from the same individual. The a priori level of statistical significance for all analyses was a two-sided value of *P*<.05. Analyses were conducted with SAS 9.4. Two alternative strategies were used in sensitivity analyses to assess the robustness of our findings to potential bias from missing data (3% or more): multiple imputation using fully conditional specification (generating and combining 10 imputed data sets) and complete-case regression analysis excluding missing observations.

## RESULTS

**Fig. 1.**
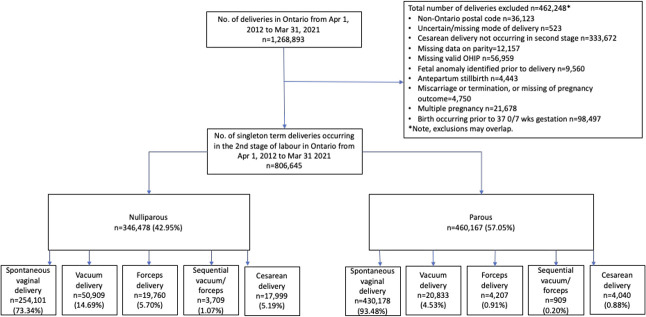
Study population by mode of delivery. OHIP, Ontario Health Insurance Plan.

There were 1,268,893 births in Ontario between April 1, 2012, and March 31, 2021. After exclusions, the study population consisted of 806,645 singleton term births in the second stage of labor (Fig. 1). Nulliparous individuals comprised 43.0% of the cohort, with 73.3% achieving spontaneous vaginal birth, 21.5% having operative vaginal birth (14.7% vacuum, 5.7% forceps, 1.1% sequential operative vaginal birth), and 5.2% having second-stage cesarean birth, resulting in a ratio of second-stage cesarean birth to operative vaginal birth of 0.24 (Appendix 2, available online at http://links.lww.com/AOG/E154). In contrast, 93.5% of parous individuals had a spontaneous vaginal birth, 5.6% had an operative vaginal birth (4.5% vacuum, 0.9% forceps, and 0.2% sequential operative vaginal birth), and 0.9% had a second-stage cesarean birth, yielding a ratio of second-stage cesarean birth to operative vaginal birth of 0.16.

*Hébert. Trends in Second-Stage Cesarean Birth. O&G Open 2025*.

**Fig. 2.**
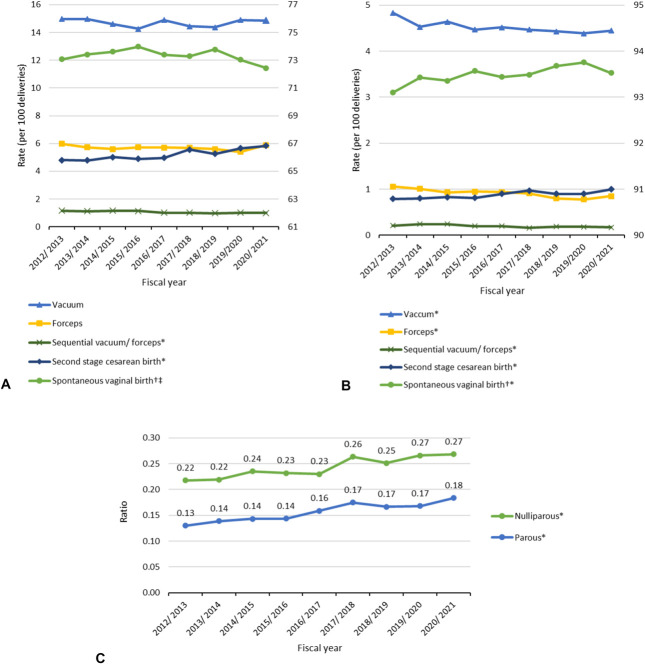
Temporal trends in mode of delivery (per 100 deliveries) among nulliparous (**A**) and parous (**B**) individuals and ratios of cesarean to operative vaginal birth (**C**) in the second stage of labor in Ontario Canada, 2012–2021. Each fiscal year ranges from April 1 to March 31 inclusively. *Cochran–Armitage test for linear trend *P*<.01. ^†^Spontaneous vaginal birth is plotted on the secondary axis. ^‡^Cochran–Armitage test for linear trend *P*<.05.

Temporal trends in the mode of delivery are shown in Figure 2 and summarized in Appendix 2, http://links.lww.com/AOG/E154. Among nulliparous individuals, the second-stage cesarean birth rate increased by 21.2%, from 4.8% to 5.8% (*P* for trend<.001); spontaneous vaginal births decreased from 73.1% to 72.4% (*P*=.025); and sequential operative vaginal births decreased from 1.2% to 1.0% (*P*<.001). Rates of vacuum and forceps delivery remained stable. The second-stage cesarean birth to operative vaginal birth ratio increased by 23.4% (0.22–0.27, *P*<.001), driven by the increase in second-stage cesarean birth rates because operative vaginal birth rates remained relatively stable. Among parous individuals, the second-stage cesarean birth rate increased by 26.6%, from 0.8% to 1.0% (*P*<.001), alongside decreases in all types of operative vaginal births: vacuum deliveries decreased by 8.1% (*P*<.001), forceps deliveries decreased by 19.8% (*P*<.001), and sequential operative vaginal births decreased by 19.0% (*P*=.002). The ratio of second-stage cesarean births to operative vaginal births increased by 40.9% (0.13–0.18, *P*<.001). Spontaneous vaginal birth rates increased slightly, from 93.1% to 93.5% (*P*<.001).

*Hébert. Trends in Second-Stage Cesarean Birth. O&G Open 2025*.

The most common primary indication for second-stage cesarean birth among nulliparous and parous individuals was obstructed labor in the second stage (59.9% and 43.2%, respectively), followed by abnormal fetal heart rate (24.4% and 31.8%, respectively) (Appendix 3, available online at http://links.lww.com/AOG/E154). Over the study period, the rate of second-stage cesarean birth attributable to abnormal fetal heart rate increased significantly for nulliparous individuals (18.5–26.3%, *P*<.001) and multiparous individuals (24.4–37.3%, *P*<.001) (Appendices 4 and 5, available online at http://links.lww.com/AOG/E154). In contrast, second-stage cesarean birth attributed to obstructed labor decreased (nulliparous individuals 66.6–57.7%, *P*<.001; multiparous individuals 51.7–39.1%, *P*<.001). The rate of second-stage cesarean birth attributable to failed operative vaginal birth also decreased among nulliparous individuals (8.1–5.9%, *P*<.001), whereas second-stage cesarean birth attributable to other maternal indications increased (1.7–3.0%, *P*<.001). For multiparous individuals, second-stage cesarean birth attributable to other maternal indications decreased (8.3–5.9%, *P*=.007).

Maternal, obstetric, and fetal–neonatal characteristics by mode of delivery are presented in Table 1 for nulliparous individuals. Imbalances between the second-stage cesarean birth and operative vaginal birth groups were observed across several variables (Appendix 6, available online at http://links.lww.com/AOG/E154). The second-stage cesarean birth group had a higher proportion of individuals aged 30 years or older and with elevated prepregnancy BMI; the operative vaginal birth group included more Black and Asian individuals. Fewer smokers were in the second-stage cesarean birth group, and missing data for other comorbidities were more common in the operative vaginal birth group. Factors such as first-trimester visit, midwife or family physician care, labor induction, intrapartum oxytocin, and neuraxial analgesia were more common in the second-stage cesarean birth group, as were macrosomia, gestational age of 41 weeks or more, and nonvertex fetal presentation. Similar imbalances were observed in parous individuals (Appendices 7 and 8, available online at http://links.lww.com/AOG/E154), although there were no meaningful differences in the distribution of age, labor induction, or intrapartum oxytocin use. The second-stage cesarean birth group also had a higher proportion of individuals with previous cesarean birth.

**Table 1.** Distribution of Maternal, Obstetric Practice, and Fetal–Neonatal Characteristics by Mode of Delivery Among Nulliparous Individuals With Singleton Term Births in the Second Stage of Labor, Ontario, Canada, 2012–2021

Characteristic	All Deliveries (n=346,478)	Spontaneous Vaginal Birth [n=254,101 (73.34)]	Vacuum [n=50,909 (14.69)]	Forceps [n=19,760 (5.70)]	Sequential Vacuum and Forceps [n=3,709 (1.07)]	SSCB [n=17,999 (5.19)]	Standardized Difference, SSCB vs OVB*
Maternal characteristics							
Age (y)							0.14
Younger than 20	15,078 (4.35)	12,670 (4.99)	1,510 (2.97)	435 (2.20)	113 (3.05)	350 (1.94)	
20–24	52,650 (15.20)	42,154 (16.59)	6,293 (12.36)	2,082 (10.54)	449 (12.11)	1,672 (9.29)	
25–29	116,913 (33.74)	87,413 (34.40)	16,474 (32.36)	6,190 (31.33)	1,193 (32.17)	5,643 (31.35)	
30–34	118,382 (34.17)	83,571 (32.89)	18,890 (37.11)	7,579 (38.36)	1,360 (36.67)	6,982 (38.79)	
35 or older	43,450-43,455 (12.54)	28,293 (11.13)	7,742 (15.21)	3,469- 3,474 (17.56-17.58)	594 (16.02)	3,352 (18.62)	
Missing or unknown	0-5 (0.00)†	0 (0.00)	0 (0.00)	0-5 (0.00-0.03)†	0 (0.00)	0 (0.00)	
Prepregnancy BMI (kg/m^2^)							0.31
Lower than 18.5	24,135 (6.97)	17,826 (7.02)	4,018 (7.89)	1,215 (6.15)	279 (7.52)	797 (4.43)	
18.5–24.9	178,181 (51.43)	130,799 (51.48)	27,032 (53.10)	10,193 (51.58)	1,921 (51.79)	8,236 (45.76)	
25.0–29.9	66,054 (19.06)	48,034 (18.90)	9,032 (17.74)	3,984 (20.16)	687 (18.52)	4,317 (23.98)	
30.0–34.9	25,385 (7.33)	18,810 (7.40)	3,107 (6.10)	1,476 (7.47)	249 (6.71)	1,743 (9.68)	
35.0–39.9	10,223 (2.95)	7,658 (3.01)	1,214 (2.38)	541 (2.74)	75 (2.02)	735 (4.08)	
40.0 or higher	5,964 (1.72)	4,447 (1.75)	677 (1.33)	340 (1.72)	55 (1.48)	445 (2.47)	
Missing or unknown	36,536 (10.54)	26,527 (10.44)	5,829 (11.45)	2,011 (10.18)	443 (11.94)	1,726 (9.59)	
Race							0.14
Asian	64,762 (18.69)	43,508 (17.12)	12,885 (25.31)	4,043 (20.46)	928 (25.02)	3,398 (18.88)	
Black	12,179 (3.52)	9,806 (3.86)	1,557 (3.06)	378 (1.91)	85 (2.29)	353 (1.96)	
White	153,968 (44.44)	114,050 (44.88)	21,038 (41.32)	9,163 (46.37)	1,554 (41.90)	8,163 (45.35)	
None of the above	12,956 (3.74)	9,576 (3.77)	1,915 (3.76)	730 (3.69)	133 (3.59)	602 (3.34)	
Missing or unknown	102,109 (29.47)	77,161 (30.37)	13,514 (26.55)	5,446 (27.56)	1,009 (27.20)	4,979 (27.66)	
Material deprivation (ON-Marg quintile)							0.08
1 (low)	65,691 (18.96)	47,949 (18.87)	9,623 (18.90)	4,088 (20.69)	667 (17.98)	3,364 (18.69)	
2	73,685 (21.27)	53,939 (21.23)	10,787 (21.19)	4,327 (21.90)	791 (21.33)	3,841 (21.34)	
3	70,971 (20.48)	51,696 (20.34)	10,750 (21.12)	3,961 (20.05)	816 (22.00)	3,748 (20.82)	
4	63,914 (18.45)	46,819 (18.43)	9,453 (18.57)	3,464 (17.53)	701 (18.90)	3,477 (19.32)	
5 (high)	67,522 (19.49)	50,033 (19.69)	9,761 (19.17)	3,708 (18.77)	688 (18.55)	3,332 (18.51)	
Missing or unknown	4,695 (1.36)	3,665 (1.44)	535 (1.05)	212 (1.07)	46 (1.24)	237 (1.32)	
Comorbidities							
Preexisting diabetes							0.08
No	338,844 (97.80)	248,839 (97.93)	49,481 (97.19)	19,305 (97.70)	3,573 (96.33)	17,646 (98.04)	
Yes	1,633 (0.47)	1,003 (0.39)	278 (0.55)	131 (0.66)	35 (0.94)	186 (1.03)	
Missing or unknown	6,001 (1.73)	4,259 (1.68)	1,150 (2.26)	324 (1.64)	101 (2.72)	167 (0.93)	
Gestational diabetes							0.09
No	321,324 (92.74)	236,410 (93.04)	46,687 (91.71)	18,281 (92.52)	3,378 (91.08)	16,568 (92.05)	
Yes	19,756 (5.70)	13,885 (5.46)	3,168 (6.22)	1,196 (6.05)	239 (6.44)	1,268 (7.04)	
Missing or unknown	5,398 (1.56)	3,806 (1.50)	1,054 (2.07)	283 (1.43)	92 (2.48)	163 (0.91)	
Preexisting hypertension							0.18
No	325,308 (93.89)	239,486 (94.25)	46,936 (92.20)	18,228 (92.25)	3,363 (90.67)	17,295 (96.09)	
Yes	2,455 (0.71)	1,712 (0.67)	389 (0.76)	174 (0.88)	25 (0.67)	155 (0.86)	
Missing or unknown	18,715 (5.40)	12,903 (5.08)	3,584 (7.04)	1,358 (6.87)	321 (8.65)	549 (3.05)	
HDP							0.21
No	324,356 (93.62)	238,272 (93.77)	47,367 (93.04)	18,375 (92.99)	3,455 (93.15)	16,887 (93.82)	
Yes	15,966 (4.61)	11,300 (4.45)	2,347 (4.61)	1,043 (5.28)	174 (4.69)	1,102 (6.12)	
Missing or unknown	6,156 (1.78)	4,529 (1.78)	1,195 (2.35)	342 (1.73)	80 (2.16)	10 (0.06)	
Smoking							0.14
No	309,399 (89.30)	225,867 (88.89)	45,732 (89.83)	17,944 (90.81)	3,262 (87.95)	16,594 (92.19)	
Yes	27,955 (8.07)	21,758 (8.56)	3,587 (7.05)	1,173 (5.94)	263 (7.09)	1,174 (6.52)	
Missing or unknown	9,124 (2.63)	6,476 (2.55)	1,590 (3.12)	643 (3.25)	184 (4.96)	231 (1.28)	
Assisted reproductive technology							0.15
No	322,016 (92.94)	237,612 (93.51)	46,462 (91.26)	17,867 (90.42)	3,297 (88.89)	16,778 (93.22)	
Yes	7,366 (2.13)	4,631 (1.82)	1,350 (2.65)	600 (3.04)	118 (3.18)	667 (3.71)	
Missing or unknown	17,096 (4.93)	11,858 (4.67)	3,097 (6.08)	1,293 (6.54)	294 (7.93)	554 (3.08)	
Obstetric practice factors							
1st-trimester visit							0.20
No	25,170 (7.26)	18,696 (7.36)	3,812 (7.49)	1,266 (6.41)	282 (7.60)	1,114 (6.19)	
Yes	292,918 (84.54)	215,448 (84.79)	42,015 (82.53)	16,483 (83.42)	2,984 (80.45)	15,988 (88.83)	
Missing or unknown	28,390 (8.19)	19,957 (7.85)	5,082 (9.98)	2,011 (10.18)	443 (11.94)	897 (4.98)	
Intrapartum admitting clinician							0.19
Obstetrician	250,701 (72.36)	179,029 (70.46)	40,018 (78.61)	15,719 (79.55)	2,942 (79.32)	12,993 (72.19)	
Midwife	48,133 (13.89)	38,270 (15.06)	4,778 (9.39)	2,076 (10.51)	378 (10.19)	2,631 (14.62)	
Family physician	34,087 (9.84)	25,517 (10.04)	4,670 (9.17)	1,381 (6.99)	296 (7.98)	2,223 (12.35)	
Other	7,841 (2.26)	5,575 (2.19)	1,438-1,443 (2.82-2.83)	579-584 (2.93-2.96)	93 (2.51)	152 (0.84)	
Missing or unknown	5,716 (1.65)	5,710 (2.25)	0-5 (0.00-0.01)†	0-5 (0.00-0.03)†	0 (0.00)	0 (0.00)	
Labor induction							0.14
No	236,377 (68.22)	177,672 (69.92)	33,547 (65.90)	12,342 (62.46)	2,339 (63.06)	10,477 (58.21)	
Yes	110,094 (31.78)	76,424-76,429 (30.08)	17,357-17362 (34.09-34.10)	7,418 (37.54)	1,370 (36.94)	7,517-7,522 (41.76-41.79)	
Missing or unknown	7 (0.00)	0-5 (0.00)†	0-5 (0.00-0.01)†	0 (0.00)	0 (0.00)	0-5 (0.00-0.03)†	
Intrapartum oxytocin							0.19
No	128,827 (37.18)	105,555 (41.54)	14,570 (28.62)	4,381 (22.17)	928 (25.02)	3,393 (18.85)	
Yes	217,651 (62.82)	148,546 (58.46)	36,339 (71.38)	15,379 (77.83)	2,781 (74.98)	14,606 (81.15)	
Neuraxial analgesia in labor							0.13
No	79,611 (22.98)	69,075 (27.18)	7,154 (14.05)	1,362 (6.89)	398 (10.73)	1,622 (9.01)	
Yes	261,723 (75.54)	181,160 (71.29)	42,951 (84.37)	18,104 (91.62)	3,249 (87.60)	16,259 (90.33)	
Missing or unknown	5,144 (1.48)	3,866 (1.52)	804 (1.58)	294 (1.49)	62 (1.67)	118 (0.66)	
Fetal–neonatal characteristics							
Gestational age (wk)							0.19
37 0/7–40 6/7	289,761 (83.63)	215,481 (84.80)	41,974 (82.45)	15,749 (79.70)	2,943 (79.35)	13,614 (75.64)	
41 0/7–41 6/7	54,809 (15.82)	37,339 (14.69)	8,640 (16.97)	3,881 (19.64)	742 (20.01)	4,207 (23.37)	
42 0/7 or more	1,908 (0.55)	1,281 (0.50)	295 (0.58)	130 (0.66)	24 (0.65)	178 (0.99)	
Presentation							0.22
Cephalic	331,567 (95.70)	244,287 (96.14)	48,253 (94.78)	18,710 (94.69)	3,451 (93.04)	16,866 (93.71)	
Noncephalic	1,827 (0.53)	697 (0.27)	214 (0.42)	200 (1.01)	33 (0.89)	683 (3.79)	
Missing or unknown	13,084 (3.78)	9,117 (3.59)	2,442 (4.80)	850 (4.30)	225 (6.07)	450 (2.50)	
Birth weight (g)							0.30
Less than 4,000	318,744 (92.00)	235,947 (92.86)	47,066 (92.45)	17,665 (89.40)	3,340 (90.05)	14,726 (81.82)	
4,000–4,499	23,469 (6.77)	15,368 (6.05)	3,253 (6.39)	1,791 (9.06)	305 (8.22)	2,752 (15.29)	
4,500 or more	2,592 (0.75)	1,496 (0.59)	336 (0.66)	232 (1.17)	46 (1.24)	482 (2.68)	
Missing or unknown	1,673 (0.48)	1,290 (0.51)	254 (0.50)	72 (0.36)	18 (0.49)	39 (0.22)	
Newborn anomaly							0.01
No	341,732 (98.63)	250,686 (98.66)	50,206 (98.62)	19,488 (98.62)	3,637 (98.06)	17,715 (98.42)	
Yes	4,746 (1.37)	3,415 (1.34)	703 (1.38)	272 (1.38)	72 (1.94)	284 (1.58)	

SSCB, second stage cesarean birth; OVB, operative vaginal birth; S, suppressed; BMI, body mass index; ON-Marg, Ontario Marginalization Index; HDP, hypertensive disorders of pregnancy.

Data are n (%) unless otherwise specified.

* Standardized difference values above 0.1 are considered indicative of a meaningful difference between groups.

† Small numbers (less than 6) were reported as ranges to ensure nonidentification.

For nulliparous individuals, factors with the strongest association with second-stage cesarean birth compared with operative vaginal birth included prepregnancy BMI of 40 or higher (aRR 1.49, 95% CI, 1.37–1.62), admission by a midwife (aRR 1.41, 95% CI, 1.36–1.47) or family physician (aRR 1.45, 95% CI, 1.39–1.50), and birth weight of 4,500 g or more (aRR 2.07, 95% CI, 1.93–2.22) (Fig. 3A). Intrapartum oxytocin (aRR 1.32, 95% CI, 1.28–1.37) and neuraxial analgesia (aRR 1.14, 95% CI, 1.12–1.23) had the highest population attributable fraction, accounting for 19.8% and 13.4% of second-stage cesarean birth cases, respectively (Appendix 6, http://links.lww.com/AOG/E154).

Fig. 3. Forest plot of maternal, obstetric practice, and fetal or neonatal characteristics associated with second-stage cesarean delivery (SSCD) compared with operative vaginal delivery (OVD) among nulliparous (**A**) and parous (**B**), singleton term births in Ontario Canada, 2012–2021. *Model adjusted for age, body mass index (BMI), ethnicity, material deprivation, preexisting diabetes, gestational diabetes, preexisting hypertension, hypertensive disorders of pregnancy (HDP), previous cesarean birth (parous only), smoking, assisted reproductive technologies (ART), first-trimester visit, intrapartum admitting clinician, labor induction, intrapartum oxytocin, neuraxial analgesia in labor, gestational age, birth weight, and newborn anomaly. ON-Marg, Ontario Marginalization Index.

**Figure:**
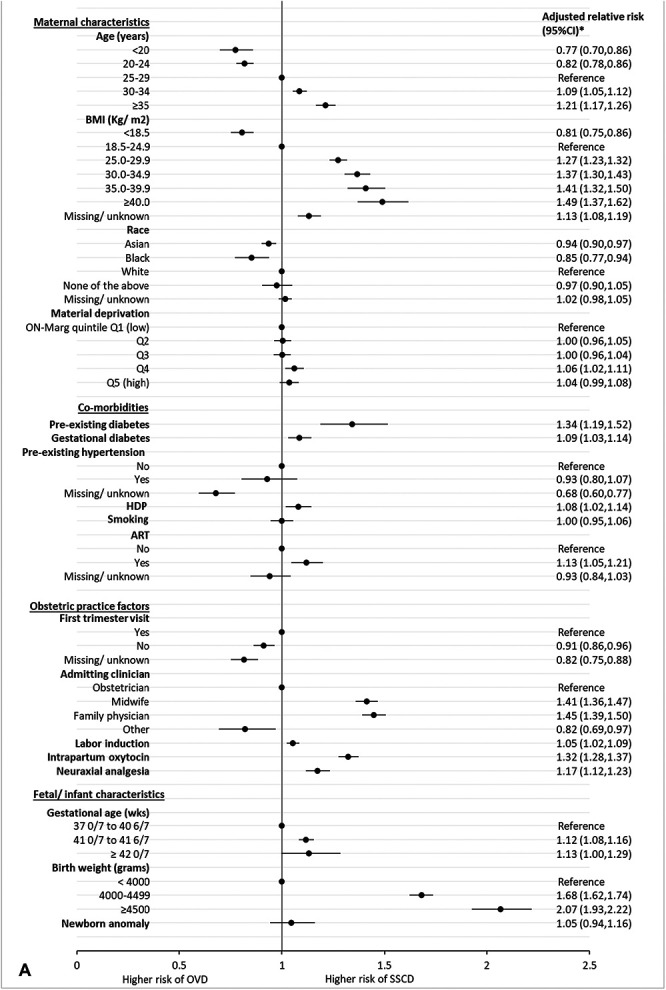

**Figure:**
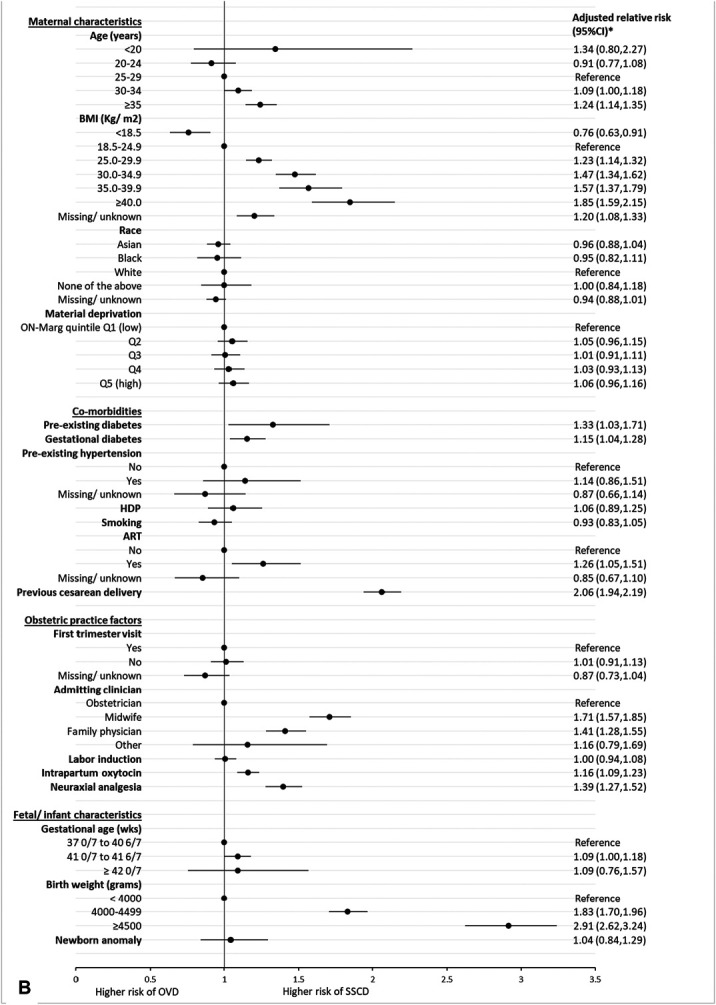
*Hébert. Trends in Second-Stage Cesarean Birth. O&G Open 2025*.

In parous individuals, significant associations with second-stage cesarean birth compared with operative vaginal birth included BMI of 40 or higher (aRR 1.85, 95% CI, 1.59–2.15), previous cesarean birth (aRR 2.06, 95% CI, 1.94–2.19), admission by a midwife (aRR 1.71, 95% CI, 1.57–1.85), neuraxial analgesia (aRR 1.39, 95% CI, 1.27–1.52), and birth weight of 4,500 g or more (aRR 2.91, 95% CI, 2.62–3.24) (Fig. 3B). Neuraxial analgesia and previous cesarean birth had the highest population attributable fractions, contributing to 24.0% and 18.0% of second-stage cesarean birth cases, respectively (Appendix 8, http://links.lww.com/AOG/E154). Analyses restricted to individuals with no missing information and models with missing values imputed yielded similar results (Appendix 9, available online at http://links.lww.com/AOG/E154).

**Fig. 4.**
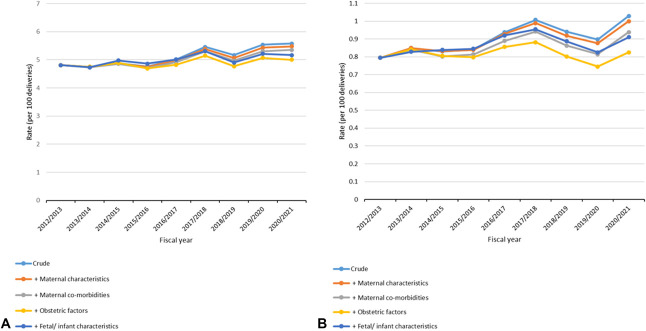
Crude rates and rates of second-stage cesarean birth sequentially adjusted for changes in individual characteristics,* comorbidities,^†^ obstetric practice factors,^‡^ and fetal or neonatal characteristics^§^ among nulliparous (**A**) and parous (**B**) individuals in Ontario Canada, 2012–2021. Each fiscal year ranges from April 1 to March 31 inclusively. *Individual characteristics included age, prepregnancy body mass index, and material deprivation. ^†^Comorbidities included preexisting diabetes, gestational diabetes, preexisting hypertension, hypertensive disorders of pregnancy, smoking, assisted reproductive technologies, and previous cesarean birth (parous only). ^‡^Obstetric practice factors included first-trimester visit, labor induction, intrapartum oxytocin, and neuraxial analgesia in labor. ^§^Fetal or neonatal characteristics included fetal presentation, gestational age, birth weight, and newborn anomaly.

The crude rate of second-stage cesarean birth increased over the study period for nulliparous and parous individuals (nulliparous individuals: RR 1.16, 95% CI, 1.08–1.24; multiparous individuals: RR 1.30, 95% CI, 1.11–1.51) (Fig. 4). Changes in maternal and obstetric practice factors are detailed in Appendices 10 and 11, available online at http://links.lww.com/AOG/E154. For both groups, rates of gestational diabetes, assisted reproductive technologies, and labor induction increased, whereas for nulliparous individuals, intrapartum oxytocin use and neuraxial analgesia also rose significantly. After sequential adjustment for individual characteristics, comorbidities, and obstetric practices, the increase in second-stage cesarean birth rates was largely attenuated (nulliparous individuals: aRR 1.04, 95% CI, 0.97–1.11; multiparous individuals: aRR 1.04, 95% CI, 0.89–1.21) (Appendix 12, available online at http://links.lww.com/AOG/E154, and Fig. 4). Obstetric factors alone explained 46.3% of the 16.0% rise in second-stage cesarean birth rates for nulliparous individuals and 48.6% of the 29.6% increase for multiparous individuals. Although maternal and obstetric risk factors increased, fetal and neonatal risk factors—including postterm gestation, macrosomia, and newborn anomalies—declined. After adjustment for these factors, the temporal trend “bounced back,” highlighting that the increase in second-stage cesarean birth rates is driven primarily by maternal and obstetric factors.

*Hébert. Trends in Second-Stage Cesarean Birth. O&G Open 2025*.

## DISCUSSION

This study found that 26.7% of nulliparous individuals and 6.5% of multiparous individuals with singleton term pregnancies had an operative delivery at full dilation, with ratios of second-stage cesarean birth to operative vaginal birth of 1:4 for nulliparous individuals and 1:6 for multiparous individuals. Both second-stage cesarean birth rates and second-stage cesarean birth to operative vaginal birth increased during the study period. Although obstructed labor was the leading indication for second-stage cesarean birth, its contribution declined and second-stage cesarean birth rates attributable to abnormal fetal heart rate increased. The strongest factors associated with second-stage cesarean birth compared with operative vaginal birth included macrosomia, history of cesarean birth, elevated prepregnancy BMI, and care by a midwife or family physician. Neuraxial analgesia, intrapartum oxytocin (nulliparous individuals), and previous cesarean birth (multiparous individuals) had the highest population attributable fractions. After adjustment for maternal and obstetric factors, the temporal increase in second-stage cesarean birth rates was attenuated, indicating that these factors largely explain the increase, with changes in obstetric practices playing a prominent role.

A recent systematic review found second-stage cesarean birth rates (2.65%) and ratios of second-stage cesarean birth to operative vaginal birth (0.19) similar to ours, but the included studies involved multiple and preterm pregnancies, limiting comparability.^18^ The primary indications for second-stage cesarean birth and the temporal increase in second-stage cesarean birth rates are consistent with other studies.^7–10,12^ However, our finding of stable forceps rates in nulliparous individuals contrasts with a decline observed in other research^2,3^ but is consistent with a study reporting a similar stabilization of forceps use among nulliparous individuals in Canada starting in 2010, after a period of decline.^21^ This trend may reflect the implementation of initiatives in Canada aimed at improving skills and training in assisted vaginal births, introduced in response to rising cesarean birth rates.^17,22^ These efforts may have played a role in halting the decline in forceps use in some regions.

The factors associated with second-stage cesarean birth compared with operative vaginal birth provide insights for future causal research, but these associations are descriptive and should not be interpreted as causal. The association between second-stage cesarean birth and elevated BMI aligns with prior research,^23^ possibly reflecting variations in labor management,^24^ higher operative vaginal birth failure,^25^ or reduced operative vaginal birth attempts.^26^ Epidural analgesia has also been associated with an increased risk of second-stage cesarean birth without an operative vaginal birth attempt,^27^ although the evidence is conflicting.^28^ The association between midwifery or family physician care and second-stage cesarean birth compared with operative vaginal birth may be the result of lower intervention rates,^29^ influencing the profile of individuals entering the second stage. In addition, because midwives and many family physicians in Ontario do not perform operative deliveries, care may be transferred to obstetricians at higher thresholds, potentially influencing decision making.

We found that increases in obstetric practices such as labor induction, intrapartum oxytocin use, and neuraxial analgesia were the strongest modifiable contributors to the rise in second-stage cesarean birth rates, despite their likely role in reducing fetal–neonatal risk factors that buffered this trend. Research demonstrates the complex relationship between these interventions and cesarean birth risk. For example, Kotaska et al^30^ highlighted the differential effect of epidural and dose of intrapartum oxytocin on cesarean birth risk. The effects of epidural analgesia and labor induction methods on fetal heart rate abnormalities—a rising indication for second-stage cesarean birth in our study—have also been emphasized in recent work.^31,32^ These findings underscore the need for a better understanding of the interactions between obstetric interventions and the indications driving the rising second-stage cesarean birth rates.

Rising second-stage cesarean birth rates suggest a potential shift away from operative vaginal births, especially in multiparous individuals, and from spontaneous vaginal births in nulliparous individuals. Although the broader effects of these trends on maternal and neonatal outcomes remain poorly understood, evidence of downstream effects of second-stage cesarean birth such as cervical insufficiency is concerning.^33^ The factors identified in this study offer valuable entry points for addressing this increase. Still, deeper exploration is needed, particularly in how these factors intersect with physician skill, risk perception, malpractice pressures, and clinical context.^2,8,34^ In addition, patient choice in the mode of delivery is paramount and deserves careful consideration. This study contributes previously unavailable information about second-stage deliveries that may be valuable for patient counseling and informed decision making.

This study is not without limitations. First, there is the potential for transcription and coding errors, common in large databases. Second, characteristics potentially associated with operative delivery, including physician experience, second-stage duration, cervical ripening, fetal position, and fetal station, could not be explored because of data limitations. Third, a significant proportion of individuals had missing data on important factors associated with the mode of delivery (eg, BMI, race), which was handled with a missing indicator approach. However, both complete-case and imputed analyses yielded similar results.

In summary, changes in obstetric practices are the primary modifiable drivers of the observed temporal increase in second-stage cesarean birth rates in Ontario, Canada. A better understanding of these practices and the indications for second-stage cesarean birth can inform targeted strategies to address these rising rates.
